# The brain-gut axis: communication mechanisms and the role of the microbiome as a neuroprotective factor in the development of neurodegenerative diseases: A literature overview

**DOI:** 10.3934/Neuroscience.2024019

**Published:** 2024-08-28

**Authors:** mgr Natalia Białoń, dr hab. n. o zdr. Dariusz Górka, mgr Mikołaj Górka

**Affiliations:** 1 Faculty of Health Sciences in Katowice, Department of Sports Medicine and Physiology of Physical Exercise, Medical University of Silesia in Katowice, 12 Medyków St., 40-752 Katowice, Poland; 2 Center for Experimental Medicine of the Silesian Medical University in Katowice, 4 Medyków St., 40-752 Katowice, Poland

**Keywords:** microbiome, brain-gut axis, neurodegenerative diseases, psychobiotics

## Abstract

The study of the brain-gut axis and its impact on cognitive function and in the development of neurodegenerative diseases is a very timely topic of interest to researchers. This review summarizes information on the basic mechanisms of gut-brain communication. We then discuss the roles of the gut microbiome as a neuroprotective factor in neurodegeneration. The gut microbiota is extremely important in maintaining the body's homeostasis, shaping the human immune system and the proper functioning of the brain. The intestinal microflora affects the processes of neuroplasticity, synaptogenesis, and neuronal regeneration. This review aims to explain changes in the composition of the bacterial population of the intestinal microflora among patients with Alzheimer's disease, Parkinson's disease, and multiple sclerosis. Abnormalities in gut microflora composition are also noted in stress, depression, or autism spectrum development. New observations on psychobiotic supplementation in alleviating the symptoms of neurodegenerative diseases are also presented.

## 1. Introduction

The gut-brain axis has been at the center of scientific interest over the past few years, having shown surprising evidence of its possible communication and influence on the central nervous system (CNS) and therefore on mental processes and behavior [1]. It represents a complex system of communication between the central nervous system and the gastrointestinal tract. This bidirectional interaction between the gut and the brain involves a variety of mechanisms, including neural, hormonal, and immunological. One of the key components of this axis, which is at the center of scientific attention, is the microbiota—a group of microorganisms that colonize the human body, formed by bacteria, fungi, archaea, protozoa, and viruses [2],[3]. In turn, the genomes of all microorganisms are referred to by the term microbiome [3]. A review of the literature indicates that the number of microorganisms inhabiting the host body exceeds the number of its cells tenfold [1],[3].

The microbiota is a complex community of microorganisms that inhabit various parts of the human body. The largest concentration of microbiota is in the gastrointestinal tract, particularly in the large intestine. The microbiota also inhabits other parts of the body, such as the mouth, nasal passages, lungs, skin, bladder, and vagina. Each of these sites is characterized by a unique composition of microbiota, adapted to the specific environmental conditions of that part of the body. The greatest diversity in terms of microbial species as well as genetic diversity is characterized by the gut microbiota (GM). Currently, the latest calculations confirm that the total mass of intestinal microbiota ranges from 1 to 2 kg [3]–[5]. By analyzing the composition of the mass of intestinal contents (200–250 g) within the colon, Daniels' (2020) showed that the bacteria representing the intestinal microbiome account for as much as about 100 g of the mass studied [6]. It is worth emphasizing that the composition of GM is characterized by great diversity and, in addition to bacteria, there are also fungi, viruses, and some protists [7]. It is undeniable that the gut microbiota demonstrates a key role in health by maintaining the body's homeostasis, creating immunity, regulating digestive processes, or synthesizing metabolites, vitamins, hormones, and neurotransmitters. There is increasing scientific evidence that the microbiome plays an important role in mental health, influencing behavior, mood regulation, and even neurodegenerative processes. The importance of the gut-brain axis is becoming increasingly recognized in the context of public health and medicine. Abnormalities in the regulation of this axis have been linked to a number of conditions, including irritable bowel syndrome (IBS), neurodegenerative diseases, and psychiatric disorders such as depression and anxiety. It is also worth referring to the role of the oral microbiota, which in recent years has been the subject of research in the development of neurodegenerative disorders [8].

The aim of this literature overview is to summarize the underlying mechanisms of gut-brain axis communication and to explore the role of the microbiome as a neuroprotective factor in nervous system regeneration. Next, we pay attention to dysbiosis of the microbiome in various organs in the development of neurodegenerative diseases and present changes in the microflora group among patients with Alzheimer's and Parkinson's disease.

## 2. The main mechanisms of brain-gut communication

### 2.1. The development of the gut microbiota

The development of the gut microbiota starts during fetal life; the later diversity and composition of the child's gut bacteria are influenced by many factors, including the gut bacteria of the pregnant parent, their diet, and their health condition [9],[10]. Childbirth plays a distinctive role in the development of the microbiota and its colonization of the human gastrointestinal tract. Natural childbirth is beneficial for microbiota development, during which the fetus, as it passes through the genital tract, is colonized by the physiological microbiota of the mother's vagina [9]. Dominguez-Bello et al. showed that the microbiota of newborns born by the natural route approximated the vaginal microbiota with a high proportion of *Lactobacilli*, in contrast to newborns born by cesarean section, whose microbiota was characterized by a higher proportion of skin bacteria, e.g., *Streptococcus* and *Staphylococcus* [11]. According to Cukrowska (2016), it is the period of fetal life and the first 1000 days of a child's life that “programs the microbiota”, demonstrating a long-term and crucial impact on human development and health [12]. Bacterial diversity increases and reaches a composition resembling the adult microbiota within the first two years of life. Contact with new foods and the microbiota of the child's environment from siblings, the immediate environment, or pets shapes the basic bacterial base [9],[13]. Significantly, the increase in hygienization of life, leading to a reduction in children's contact with microorganisms of human and animal origin, as well as the overuse of antibiotics during pregnancy and the first two years of life, contribute to disrupting the development of the microbiota. This results in an abnormal formation of immune processes, which can lead to an excessive immune response to pollen or dust mites (allergies) or own tissues (autoimmune diseases) [14],[15]. The composition of microorganisms varies and depends on normal or disturbed intestinal physiology [16]. There are several factors that contribute to dysbiosis, defined as an imbalance of the intestinal microbiota, including an abnormal diet [17],[18], the onset of disease, or too many pathogens. It is noteworthy that prolonged stress, impaired mental state, or chronically experienced anxiety can also contribute to a change in the composition of the microbiota. A study by Foster et al. (2017) on a laboratory animal model exposed to stressors confirmed that stress disrupts the abundance of microorganisms populating the gut [19]. One of the first studies on the effect of stress on the diversity of microorganisms in the gut was conducted in 1974 by Tannock and Savage. In mice that were deprived of access to food (thus inducing a stressful situation), the gut was dominated by an *Escherichia coli* strain, and the abundance of *Lactobacillus bacteria* decreased compared with mice that were left with constant access to food [20]. Bailey et al. introduced a different type of stress by placing mice in a cage with an aggressive rodent of the same species. They observed a change in the composition of the microbiota: Mice exposed to the stressor had more *Clostridium and Roseburia* bacteria and fewer *Bacteroides and Parabacteroides* compared to the gut microbiome of control mice [21]. The figure below provides an overview of the factors influencing the composition of the species diversity of the gut microbiota.

**Figure 1.**
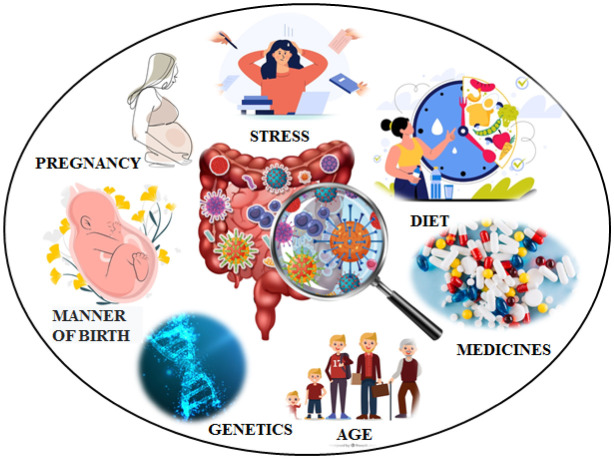
Factors influencing the composition of species diversity of intestinal microflora.

In order to maintain homeostasis as well as proper development of the organism, microbiota-gut-brain communication takes place in a continuous and bidirectional manner. There are various pathways of communication, including through the immune system, tryptophan metabolism, the vagus nerve, the enteric nervous system, and microbial metabolites such as short-chain fatty acids (SCFAs) [22].

### 2.2. The vagus nerve pathway as a direct route of communication between the gut and the brain

The direct route of communication between the gut and the brain is the tenth cranial nerve, termed the vagus nerve (*Latin nervus vagus*). Its name derives from the Latin word vagus, meaning “wandering”, as a result of its extensive innervation to receive information from various visceral organs [23]. The vagus nerve consists of 80% afferent and 20% efferent fibers, which are involved in the transmission of information from the gastrointestinal, respiratory, and cardiovascular systems [24],[25]. A special role is attributed to the nucleus tractus solitarius (NTS), which is located in the medulla oblongata. It represents the first structure to receive a signal from the gastrointestinal tract via the vagus nerve [26]. To confirm whether communication occurring through the vagus nerve is one of the main tracts of the gut-brain axis, a vagotomy procedure, i.e., cutting the vagus nerve, was performed on a group of rodents. This procedure resulted in the appearance of anxiety-like behavior and reduced neurogenesis in the dentate bend of the hippocampus [22].

### 2.3. Therapeutic potential of vagus nerve stimulation (VNS) in the treatment of neurodegenerative disorders

The known anatomical connections of the vagus nerve nuclei have provided the theoretical rationale for experimental and clinical research into the use of peripheral electrical stimulation of the vagus nerve to interfere with and modify the function of those areas of the brain responsible for emotions and mood, ultimately to produce antidepressant effects. Vagus nerve stimulation also shows wide therapeutic potential in the treatment of various neurological or degenerative diseases. VNS has been approved by the Food and Drug Administration (FDA) as an alternative treatment for epilepsy, treatment-resistant depression, migraines, or cluster headaches. There have also been promising results in the treatment of neurodegenerative diseases, including Parkinson's disease, autism spectrum disorders, traumatic brain injury, and stroke [27]. Recent studies have confirmed that VNS can inhibit inflammation, promote neuroprotection, help maintain the integrity of the blood-brain barrier, and, interestingly, transmit signals from the gut flora to the brain [28].

The use of VNS as an anti-inflammatory agent in neurological disorders is emphasized below. Diseases of the central nervous system are usually accompanied by inflammation, and excessive inflammatory responses may contribute to neuronal and glial cell death. Bonaz et al, using VNS for a period of 5 days (1 mA, 5 Hz, with a pulse width of 500 qs) on a rat model of Crohn's disease characterized by inflammatory bowel disease, confirmed that VNS reduces the extent of weight loss and levels of inflammatory markers such as TNF-α and interleukin-1 β [29]–[30]. VNS inhibits inflammation through three main mechanisms, namely an increase in cortisol levels along the HPA axis, the binding of acetylcholine to the α7nAChR receptor (this receptor is found in macrophages and microglia, playing a key role in reducing inflammation during VNS treatment) and, through splenic sympathetic nerves, affecting lymphocytes and inhibiting inflammatory responses [28]. What is interesting is that there have also been studies showing that patients with Alzheimer's disease achieved cognitive improvement after one year of VNS therapy [31]. With the development of medical technology, the use of VNS has been expanded to treat more diseases of the central nervous system [32]–[34].

In order to understand the mechanism of gut-brain communication, it is worth noting the presence of enteroendocrine cells (EECs), located in the epithelial cell layer. This is a group of sensory cells capable of sensing changes related to the influx of nutrients into the gut, metabolites of bacterial processes, and responding to them by emitting signaling molecules that include neurotransmitters or hormones. In 2018, a study was published that confirmed that EEC cells contain protrusions that form connections with the vagus nerve [35]. These cells distinguish the physical and chemical properties of the food consumed, modulate gastrointestinal function via the vagus nerve, and rapidly communicate information to the brain about the food consumed [1]. Crucially, Kaelber et al. proved that the neurotransmitter in this communication is glutamate, and this discovery was the first evidence of direct gut-brain communication, which was termed the neuroendocrine circuit [35].

### 2.4. Indirect communication routes

There are also indirect communication pathways between the microbiota and the central nervous system, which include the autonomic nervous system, the immune system, and the endocrine system [36]. The gut microbiota influences the activity of the vagus nerve by acting directly on the immune system [37],[38] by producing SCFAs or by regulating the secretion of gut hormones that affect the central nervous system [39]. It is confirmed that SCFAs modulate key bodily functions, including processing nutrients, maintaining energy homeostasis, and influencing intestinal motility to regulate intestinal homeostasis. Recent findings outline the key function of butyric acid, which has important intestinal and immune regulatory functions [40]. In addition, it is accepted that SCFAs have an important effect on the regulation of the gut-brain axis, exhibit anti-inflammatory properties, regulate the production of serotonin and gut hormones, and influence brain function by regulating microglia activity [1]. SCFA deficiency contributes to disorders such as inflammatory bowel disease, colorectal cancer, and cardiometabolic disorders. Short-chain fatty acids are metabolites, products of the fermentation process of carbohydrates and fiber carried out by the bacteria that make up the human intestinal microflora. The synthesis of SCFAs is influenced by specific foods, dietary supplements, or prebiotics [41].

A key aspect of human health is the link between the microbiota and the immune system. A review of the literature confirms that SCFA produced by *Clostridia* is involved in the activation and proliferation of Treg, or regulatory T cells, which maintain a key role in balancing the immune system. Treg have important functions in the prevention of autoimmune diseases by suppressing inflammatory reactions [42],[43]. In patients with inflammatory bowel disease, a direct effect of propionate (a microflora metabolite) on T cells has been noted, inhibiting the production of interleukin-17 (IL-17) [44]. Bile acids, which initiate the activation of farnesoid X receptor (FXR) and G1 protein-coupled bile acid receptor (GPBAR1), are also involved in maintaining intestinal immune function [45]. These receptors play important roles in the interaction between the gut microbiota and the immune system. Activation of FXR in the gut plays an important role in protecting against damage to the intestinal mucosa and also suppresses inflammation. In addition, FXR affects innate immune cells—macrophages and dendritic cells. By regulating the production of pro-inflammatory cytokines, FXR maintains immune balance in the gut. GPBAR1, in turn, plays an important role in modulating the immune response and controlling inflammation. Importantly, activation of GPBAR1 by bile acids leads to the production of anti-inflammatory cytokines and inhibits the secretion of pro-inflammatory cytokines. The importance of the gut microbiota in the development of inflammation is highly relevant, as the microflora affects the composition and circulation of bile acids, which can modulate the immune and inflammatory response in the gut [45].

It is worth noting the importance of SCFAs in the development of inflammation in the brain and their link to neurodegenerative diseases. SCFAs affect inflammation in the brain through a variety of mechanisms, including the secretion of butyrate, which has anti-inflammatory effects and can reduce the activity of microglia—the cells responsible for the inflammatory response in the brain. Excessive microglia activity leads to chronic inflammation, the basis for the development of neurodegenerative diseases [46]. Hoogland et al (47) conducted an experiment on a mouse animal model, demonstrating that injection of LPS within the peripheral nervous system, as well as heat-killed or live pathogens, develops an immune response in the rodent brain through activation of microglia. Microglia activation is associated with neuroinflammation and increased levels of TLR2, TLR$, TNFα, and IL-1β [47].

In order to assess the effect of specific bacterial populations on behavior, mouse animal models deliberately devoid of microorganisms, so-called sterile mice, are used. A review of the literature shows that the development of sterile mice proceeds in a different way compared to mice raised under standard conditions. Higher levels of stress and anxiety were observed in sterile animals, which were characterized by excessive activation of the hypothalamic-pituitary-adrenal axis (HPA), referred to as the stress axis. In addition, memory and learning difficulties appeared in these animals [48]. In turn, biochemical studies have shown reduced levels of brain-derived neurotrophic factor (BDNF), a determinant of neurogenesis and synaptogenesis, affecting cognition [49],[50]. Other evidence supporting the effect of gut bacteria on cognitive function is provided by the results of Mao et al. (2013) and De Vader et al. (2018). In sterile mice, a lower number of neurons in the enteric nervous system and a deficit in sensory signaling were observed, whereas restoration of the gut microbiota in mice restored normal gut physiology [1],[51],[52].

## 3. Changes in the microbiome as a known factor in the development of neurodegenerative diseases

Changes in the composition of the bacterial population of the intestinal microflora may correlate with the development of neurodegenerative diseases, which include Alzheimer's disease, Parkinson's disease or multiple sclerosis. Neurodegenerative diseases are defined in the literature as progressive and irreversible degeneration of nervous tissue, the cells of which die as a result of degenerative processes. A disturbed composition of the gut microbiota may also play an important role in the pathogenesis of autism spectrum disorders, anxiety, depressive behavior, or other physical disorders [53]. Neurodegenerative diseases are defined in the literature as progressive and irreversible degeneration of nervous tissue whose cells die as a result of degenerative processes. The literature on changes in the composition of the intestinal microflora and its importance in the development of neurodegenerative diseases is briefly reviewed below.

### 3.1. Alzheimer's disease

Alterations in the composition of the gut microbiota in Alzheimer's disease (AD) have been reported in the literature, with disrupted microbial composition contributing to the disease. AD is the most common cause of dementia among the elderly and accounts for 30–70% of all dementing diseases. It is characterized by irreversible, progressive cognitive and behavioral impairment accompanied by memory loss [54]. It is classified as a neurological disease, characterized by extensive neuronal loss in the brain with subsequent atrophy of the hippocampus and cortex [55],[56]. The neuropathological picture shows cortical atrophy, neurofibrillary tangles (NFT) degeneration associated with hyperphosphorylated tau protein (p-tau) that aggregates intracellularly, and extracellular senile plaques resulting from the deposition of insoluble forms of Aβ peptide in the brain [54],[57].

Patients with cognitive impairment are characterized by a poorer bacterial microflora composition; there is a decrease in *Firmicutes*-type bacteria (which include *Clostridium*) and an increase in *Bacteroides-*type microorganisms [58]. Another study showed that Alzheimer's patients with chronic *Helicobacter pylori* infection in the stomach struggle with more advanced cognitive impairment compared to patients who tested negative for the bacterium [59]. Chen et al. showed that amyloid produced by *Escherichia coli* bacteria of the *Enterobacteriaceae* family increases alpha-synuclein (a pathological protein that causes compression and restricted exchange of substances between blood and cells) [60]. This leads to a higher risk of Alzheimer's disease through interaction in cell-to-cell signaling pathways and induction of pro-inflammatory cytokines. It is worth noting, in this context, the presence of the blood-gut barrier, which has the important function of selectively absorbing nutrients while protecting against the penetration of harmful molecules from the intestinal lumen. The tightness of the blood-gut barrier plays a major role in explaining the effect of bacterial amyloid on the development of amyloid in the brain in people with Alzheimer's disease. As amyloid produced by microorganisms enters the blood from the gut and then into the brain, it increases inflammation in neural tissue [61]. This process indicates a strong interaction between the gut microbiome and brain neural tissue. Bacterial amyloid causes abnormal folding of proteins such as alpha-synuclein, which also plays a role in the pathogenesis of Alzheimer's disease [62]. Neuropathological studies of the hippocampus in patients with Alzheimer's disease have shown an increase in the presence of lipopolysaccharides, a type of bacterial endotoxin that is part of the cell membrane of Gram-negative bacteria such as *Escherichia coli* and *Bacteroides fragilis*. These lipopolysaccharides are thought to be involved in the development of the inflammatory response in the brain, and their amount was directly proportional to the severity of cognitive impairment [60]. In addition, bacterial metabolites play a role in the pathogenesis of Alzheimer's disease and short-chain fatty acids, such as butyric acid, valeric acid, and acetic acid, and demonstrate strong effects on the accumulation of amyloid and tau protein in the brain. SCFAs can cross the blood-brain barrier, negatively affecting microglia function [63]. Using a mouse animal model of the APP/PS1 line (transgenic mice carrying mutated human genes that cause Alzheimer's disease) and analyzing the species composition of their gut microbiota, significant variations were observed within the abundance of bacterial groups such as *Proteobacteriaceae, Verrucomicrobiaceae, Bifidobacteriaceae, Erysipelotrichaceae, Prevotellaceae, Bacteroidaceae, and Rikenellaceae*. In mice, dysbiosis was present long before pathological findings in the form of amyloid plaques [64]. Li et al. conducted an experiment on the differences in the microbiome between Alzheimer's patients and a control group of healthy people. By comparing the blood and fecal parameters of individuals from both groups, they identified, with a sensitivity of 93%, individuals with Alzheimer's disease who had a markedly reduced level of fecal bacterial flora diversity compared to the mild cognitive impairment and healthy groups. As the disease progressed, there was a gradual increase in the abundance of bacteria, i.e., *Gammaproteobacteria, Enterobacteriaceae, and Enterobacteriaceae* [65].

### 3.2. Parkinson's disease

Over the last few years, research interest in assessing the influence of the gut microbiota and its composition in the development of Parkinson disease (PD) has particularly increased. PD is a neurodegenerative disease characterized by resting tremor and reduced motor function. These symptoms are associated with atrophy of black matter cells in the midbrain and a reduction in the amount of dopamine secreted in striatal structures [1]. Twenty years ago, there was a theory that Parkinson's disease (PD) could start in the gut. Many recent studies seem to support this theory. As an example, a study in which the microbiota of people with PD was transplanted into sterile mice showed the appearance of symptoms of movement disorders characteristic of patients with Parkinson's disease in these animals [22]. A protein implicated in the development of Parkinson's disease is alpha-synuclein (ASyn) in its pathological form, which accumulates in the gut in the early stages of the disease, causing gastrointestinal dysfunction and abnormal intestinal peristalsis [66]. Kim et al. showed that the vagus nerve is the pathway through which ASyn travels to the brain [67]. Subsequently, aggregation of alpha-synuclein in the brain leads to activation of microglia, the growth of which determines the production of pro-inflammatory cytokines. Studies in a mouse animal model of Parkinson's disease have shown that the above process leads to neurons [68]. In addition, lipopolysaccharide present in the cell walls of Gram-negative bacteria enhances the accumulation of ASyn protein deposits [22] and induces inflammation, leaky gut, and activation of pro-inflammatory cytokines [69]. Patients with PD have a leaky gut, which causes endotoxins such as LPS to enter the bloodstream and be transported deep into the body. There have also been reports indicating differences in the composition of the microbiota of people with PD compared to that of the healthy population. Significantly elevated bacterial levels were observed in the *Enterobacteriaceae*, whose presence positively correlates with postural and gait disturbances in Parkinson's disease patients. Another study showed that the bacterial counts of *Clostridium IV*, *Aquabacterium, Holdemania, Sphingomonas, Clostridium XVIII, Butyricicoccus, and Anaerotruncus* were elevated in PD patients [22]. Marizzoni et al (2017) showed that the intestinal tract of PD patients had significantly less *Prevotella bacteria* (by 77.6% compared to the control group). These bacteria are responsible for mucus synthesis and SCFA production. A reduction in their numbers in the gastrointestinal tract may result in increased intestinal permeability and exposure of the body to constant exposure to bacterial endotoxins [70], while Perez-Pardo et al. (2017) showed that *Prevotella* is responsible for the production of vitamins B1 (thiamine) and B9 (folic acid) [71].

### 3.3. Multiple sclerosis

Studies assessing the composition and diversity of the gut microbiota are gaining increasing interest in the context of the pathogenesis and treatment of multiple sclerosis (MS). Associations between changes in the gut microbial flora and the development of MS are currently being explored. A review of the literature confirms that the state of the microbiota may predispose to MS but may also be a consequence of the disease [72]. There is a decrease in the gut bacteria *Firmicutes* and *Bacteroidetes*, which are responsible for the production of short-chain fatty acids, in patients with neurological changes specific to MS disease [73]. There is also a reduction in the numbers of *Lactobacillus, Faecalibacterium, Bacteroides*, and *Prevotella*, and additionally an increase in *Enterobacteriaceae* and *Akkermansia* bacteria, which are pro-inflammatory [72],[73]. Current findings suggest the use of probiotic therapy, fecal microbiota transplantation, or dietary changes to alleviate the development of MS symptoms [74].

Abnormalities in the composition of the gut microbiota are also noted in stress, depression, or the development of the autism spectrum. The literature confirms that there is a strong link between the composition of the gut microbiome and the development of psychiatric disorders such as depression and anxiety [75]–[77]. Patients diagnosed with depression were characterized by a different composition of the gut microbiome compared to patients in the control group. A 2019 study, conducted by Valles-Colomer et al., used 16S rRNA genetic sequencing to analyze fecal samples from 1070 individuals to describe the content and diversity of their gut microbiota. The aim of the experiment was to assess the prevalence of specific bacterial strains as well as their correlation with indicators of depression and quality of life. Among patients diagnosed with depression, *Dialister* and *Coprococcus* spp. bacterial populations were less abundant, while the presence of SCFA-producing *Faecalibacterium* and *Coprococcus* correlated with higher scores on the quality-of-life scale. It was further assessed that quality of life also correlates positively with dopamine metabolites (3,4-dihydroxyphenylacetic acid) produced by gut microbes. These findings demonstrate that the gut microbiota plays an important role in shaping affective processes [78].

Dash et al. also confirmed the link between depressive disorders and changes in intestinal bacterial flora. In patients with depression, they found a reduced number of Bacteroidetes-type bacteria with an increased number of *Alistipes* species belonging to Bacteroidetes [79]. An increase in the number of *Alistipes* species bacteria was also observed in chronic fatigue syndrome [80]. In addition, a diverse microbial profile is also noted among patients with autism spectrum disorder (ASD), which is defined as a neurodevelopmental disorder characterized by difficulties in communicating, forming social bonds, interpreting emotions (their own and other people's), and manifesting rigid behavioral patterns [1]. It is noteworthy that up to 70% of children with ASD have been observed to have gastrointestinal disorders, which may infer that the functioning of the gut microbiota plays a significant role in the etiology of the disorder [81]. A different composition of the gut microbiota in autism spectrum disorder was also observed by Strati et al.: Among individuals with ASD, the ratio of Firmicutes to Bacteroidetes bacteria was disturbed, due to a reduction in the Bacteroidetes population compared to the control group [82].

## 4. Relationship between dysbiosis of the microbiota of other body organs and the pathogenesis of neurodegenerative diseases

### 4.1. The relationship between oral microbiota dysbiosis and the pathogenesis of neurodegenerative diseases

In recent years, growing interest in the oral microbiota in the context of its impact on body homeostasis, general health, and the development of neurodegenerative diseases has resulted in new research. The oral microbiota, located within two regions (on the hard surfaces of teeth, including dentures, and the soft tissues of the oral mucosa), constitutes the second largest microbial community in the human body, comprising Actionobacteria, Bacteroidetes, Firmicutes, Fusobacteria, and Proteobacteria [83],[85]. The primary functions of the oral microbiome include regulating the immune system and blood pressure, reducing free radicals and also preventing caries. In addition, they support the remineralization and demineralization of enamel and also contribute to the regulation of the digestive system by being responsible for the metabolism of nutritional products. Deteriorated periodontal status and poor oral hygiene are more frequently observed among patients with psychiatric disorders [86]. In addition, psychiatric disorders increase the risk of caries, with a consequent increase in cavities and missing teeth [87]. The results of a study by Coelho et al. showed that the prevalence of periodontitis in patients with mental disorders is 1.45 times higher compared to healthy individuals [88]. The mechanism linking the oral microbiota in patients with psychiatric disorders is reviewed below.

One of the key mechanisms linking dysbiosis of the oral microbiota to neurodegenerative diseases is neuroinflammation. The results of a study by Domina et al. showed that the pathogenic bacteria *Porphyromonas gingivalis* found in the oral cavity produce toxic proteases termed gingipains, which have been identified in the brains of patients with Alzheimer's disease. For this purpose, 44-week-old mice were orally infected with *Porphyromonas gingivalis* every other day for six weeks. PCR analysis then showed the presence of bacteria in the brain of all mice after oral infection. The levels of toxic gingipains correlated with the presence of tau protein and ubiquitin [89]. These bacteria induce an inflammatory response that leads to neuronal damage and contributes to the pathologies characteristic of neurodegenerative diseases. Another bacterium associated with periodontal disease that has also been linked to neurodegenerative processes is *Treponema denticola*. Traces of spirochetes of this bacterium have been detected in the brains of Alzheimer's patients. Its presence activates the inflammatory pathway in the brain and also directly affects the degradation of neural tissue. *Treponema denticola* has the ability to invade gum tissue and deeper periodontal structures. Due to its motility and the presence of proteolytic enzymes such as proteases and fibrinolysins, the bacterium has the ability to enter the bloodstream. When vascular leakage occurs in peripheral tissues, microorganisms can potentially be transported from the damaged plaque into the bloodstream, thus reaching various organs and causing systemic inflammation [90]. The presence of *Treponema denticola* in the brain can lead to chronic neuroinflammation by activating microglia, leading to the production of pro-inflammatory cytokines such as interleukin 1β (IL-1β), a key mediator of the inflammatory response, or TNF-α, associated with inflammatory processes. *Treponema denticola* damages peripheral axons in the periodontal ligament, leading to disruption of axonal transport, altered mitochondrial migration, and consequent neuronal apoptosis. Chronic activation of microglia and continuous inflammation lead to neuronal damage and acceleration of neurodegenerative processes [90].

It is worth noting that the literature presents an association of oral dysbiosis in the development of neurodegenerative diseases. The presence of microorganisms that may contribute to the development of these diseases has been reported in the brains and cerebrospinal fluid of patients with Alzheimer's disease or Parkinson's disease [91]–[93]. Interestingly, a study by Emery et al. confirmed the presence of oral bacteria in brain samples of patients collected post-mortem. The bacteria were located in regions associated with Alzheimer's and Parkinson's disease pathology. It is noteworthy that the composition of bacteria in the brain differed from the microbial population in blood samples [94].

The literature also provides numerous pieces of evidence highlighting the role of the oral microbiota in Parkinson's disease [83],[95]. Significant differences in the composition of the oral and gut microbiome have been observed in PD patients compared to healthy controls [96]. A correlation between the types of bacteria in the oral cavity and the severity of depression and anxiety among patients with Parkinson's disease has also been reported [96],[97].

### 4.2. The skin microbiome in the pathogenesis of neurodegenerative diseases

Despite being one of the most important areas populated by the microbiota, the skin, due to the presence of millions of bacteria, fungi, and viruses in its composition, shows a limited influence on the development of neurodegenerative diseases compared to the gut, mouth, or brain. Indeed, it is known that the skin microbiota influences the immune system, and the body's general state of inflammation, which in turn indirectly affects mental health. However, recent studies support its potential role in the development of neuropsychiatric disorders through its ability to influence the gut microbiota [83],[84],[98],[99]. It is worth citing a study by Hermes et al. in which significant correlations were noted between an individual's body mass index and *Lactobacillus* [100]. On the other hand, a study by Arikan et al. conducted on a group of 103 patients with Parkinson's disease [101] found an association between the axillary microbiota and cognitive impairment. The results of these studies suggest a likely role for the skin microbiota in cognitive impairment.

It is worth noting that in patients with psoriasis, depression is a common psychiatric symptom [102],[103]. A review of the literature confirms the existence of a skin-microbiota-brain axis in the co-occurrence of depression in patients with chronic wounds [104]–[106]. Furthermore, dysbiosis of the skin and gut microbiome through the development of inflammatory and immune mechanisms may be a factor in the development of psoriasis [107]. Shinno-Hashimoto showed a correlation between the skin microbiota and the gut microbiota, which is a presumed bidirectional communication between the two [108].

There is no doubt that the influence of the skin microbiota in the development of neurodegenerative diseases is an area that requires further research.

### 4.3. The nasal microbiome

The nasal cavity harbors a diverse community of microorganisms that determine the homeostasis of the nasal mucosa and the overall functioning of the immune system [109]–[111]. The literature shows that there is an association between dysbiosis of the nasal microbiome and the occurrence of various health problems [84],[109],[111]. The nasal microflora forms complex connections with the nervous system through the nose-brain pathway. Microorganisms and their toxins can migrate from the nose to the brain via the olfactory nerve, which can potentially develop inflammation, contributing to the development of neurodegenerative diseases [83],[112]. Although the nasal microbiome is the least studied compared to other regions of the microbiome, there is growing interest in its role in neurological diseases.

## 5. Microbiome as a neuroprotective factor in cognitive functioning

The relationship of the gut microbiota to cognitive functioning, i.e., learning, attention, or memory, is at the center of researchers' research. Experimental results suggest that psychobiotic therapy benefits mental health and cognitive function by interacting with gut bacteria [113]. Psychobiotic supplementation can affect the composition of the microbiota, increase intestinal tightness, and reduce inflammation affecting the slowing of the neurodegenerative process [71]. Probiotic therapy positively correlates with the maintenance of normal intestinal barrier function and stimulates the immune system to initiate anti-inflammatory processes. The literature presents growing evidence confirming the role of probiotic strains in modulating the microbiota-gut-brain axis. Snigdha et al. emphasized that psychobiotic supplementation leads to significant improvements in cognitive functions, including working memory, and reduces symptoms of anxiety and depression. Furthermore, psychobiotics influence neurochemical pathways associated with neurotransmitters, reducing brain inflammation and enhancing neuroplasticity. It is also worth noting the differences in response to psychobiotics depending on gender and life stage, suggesting that the effectiveness of probiotic therapy may vary across different demographic groups [114].

Supplementation of selected bacterial strains and their effects on brain, cognitive function, and emotions are discussed below.

**Table 1.** Representation of selected bacteria and their impact on the brain, cognitive function and emotions.

Bacterial strain	Duration and effect of supplementation	Source
*Lactobacillus helveticus*	Duration of supplementation: 12 weeks. Among healthy older adults, improvements in attention and working memory were noted in tests.	[115]
*Bifidobacterium longum* 1714	Slight improvement in visual-spatial memory performance in a word memorization task.	[116]
*Bifidobacterium longum* 1714	Significant improvement in sleep length and quality during exam stress. It alleviates the symptoms of depression by modulating synaptogenesis.	[117],[118]
*Lactobacillus plantarum* DR7	Duration of supplementation: 12 weeks. Improved memory, attention, associative learning.	[119]
*L. rhamnosus* (JB-1)	Decreased stress–related behaviors,MikołajIt restores neurochemical balance in the brain, regulates mood disorders in a rat model of chronic stress, and enhances the expression of proteins involved in the activation and maturation of nerve cells. Additionally, it regulates myelination and the process of neurogenesis in a mouse model.	[120]–[122]
*L. plantarum* 90sk and *B. adolescentis* 150	Reduced depressive-like behavior	[123]
*Lactobacillus helveticus (R0052)*	As a result of supplementation, an increase in SCFAs and acetic acid was observed, along with higher concentrations of anti-inflammatory cytokines IL-6 and IL-10 (p < 0.001) and a decrease in the pro-inflammatory cytokine TNF-α.	[124]
*Bifidobacterium breve (A1)*	Inhibition of brain atrophy progression.	[125]

## 6. Conclusion and future perspective

A review of the literature shows that over the past few years, the importance of the gut microbiota, the mechanisms of communication with the brain, and the supplementation of psychobiotics has received a lot of attention, putting it at the center of scientific research. An imbalance of the microbiome contributes to the development of neurodegenerative diseases and cognitive impairment. Moreover, the known biochemical substances synthesized by bacteria are involved in maintaining the body's homeostasis, the physiology of the digestive system, the regulation of the immune system, and the work of the central nervous system. In addition, it is worth delving into the importance of the microbiome in the regeneration of the peripheral nervous system. It is known that bacteria synthesize SCFAs, which stimulate the secretion of BDNF, which plays a special role in peripheral nerve regeneration.

In conclusion, the results of future scientific research may allow the development of optimal doses of supplemented psychobiotics and thus effectively support brain function. Dietary or pharmacological intervention with psychobiotic therapy may be a promising pathway for the treatment and prevention of neurodegenerative diseases.
